# Unraveling the Intoxicating and Therapeutic Effects of Cannabis Ingredients on Psychosis and Cognition

**DOI:** 10.3389/fpsyg.2020.00833

**Published:** 2020-05-14

**Authors:** Marco Colizzi, Mirella Ruggeri, Sagnik Bhattacharyya

**Affiliations:** ^1^Section of Psychiatry, Department of Neurosciences, Biomedicine and Movement Sciences, University of Verona, Verona, Italy; ^2^Department of Psychosis Studies, Institute of Psychiatry, Psychology and Neuroscience, King’s College London, London, United Kingdom

**Keywords:** Δ-9-tetrahydrocannabinol, cannabidiol, endocannabinoid system, psychosis, cognition

## Abstract

Research evidence suggests a dose–response relationship for the association between cannabis use and risk of psychosis. Such relationship seems to reflect an increased risk of psychosis not only as a function of frequent cannabis use, but also of high-potency cannabis use in terms of concentration of Δ-9-tetrahydrocannabinol (Δ9-THC), its main psychoactive component. This finding would be in line with the evidence that Δ9-THC administration induces transient psychosis-like symptoms in otherwise healthy individuals. Conversely, low-potency varieties would be less harmful because of their lower amount of Δ9-THC and potential compresence of another cannabinoid, cannabidiol (CBD), which seems to mitigate Δ9-THC detrimental effects. A growing body of studies begins to suggest that CBD may have not only protective effects against the psychotomimetic effects of Δ9-THC but even therapeutic properties on its own, opening new prospects for the treatment of psychosis. Despite being more limited, evidence of the effects of cannabis on cognition seems to come to similar conclusions, with increasing Δ9-THC exposure being responsible for the cognitive impairments attributed to recreational cannabis use while CBD preventing such effects and, when administered alone, enhancing cognition. Molecular evidence indicates that Δ9-THC and CBD may interact with cannabinoid receptors with almost opposite mechanisms, with Δ9-THC being a partial agonist and CBD an inverse agonist/antagonist. With the help of imaging techniques, pharmacological studies *in vivo* have been able to show opposite effects of Δ9-THC and CBD also on brain function. Altogether, they may account for the intoxicating and therapeutic effects of cannabis on psychosis and cognition.

## Introduction

Public interest in cannabis has increased over the last decade for several reasons. First, cannabis is the most popular recreational drug, and its use has increased over time, with population data estimating around 200 million users worldwide (National Academies of Sciences, 2017). Second, because of ongoing decriminalization and legalization policies (Hall and Lynskey, 2016; Hasin et al., 2017), low-potency cannabis varieties have come on to the market as non-medicinal products with recreational and self-medication purposes. These preparations have a legally established limit of Δ-9-tetrahydrocannabinol (Δ9-THC), the main psychoactive ingredient of cannabis, ranging from 0.2 to 1% across countries (Small, 2015). Such non-medicinal products contain mainly cannabidiol (CBD), a non-intoxicating cannabinoid (Freeman et al., 2019), at doses far lower (e.g., 25 mg) than those ever used in human therapeutic trials (e.g., 150–1,500 mg/d) (Davies and Bhattacharyya, 2019). Some countries have questioned the safety of these so-called “cannabis light” varieties (Sachs et al., 2015), because of wide variability in cannabinoid profiles in the absence of standardized regulations (Pavlovic et al., 2018). Third, in the illicit market, the part of the cannabis plant with the highest Δ9-THC content is selected to amplify its intoxicating effects (Small, 2015), with the result that illicit cannabis potency, indexed as Δ-9-THC concentration, has increased over the last 30 years (Cascini et al., 2012). This leads to a fourth reason for public focus, as accumulating evidence indicates a dose–response relationship between increasing Δ9-THC exposure and harm attributable to or related to cannabis use (Freeman et al., 2018). Neuropsychiatric and substance use disorders account for the larger part of the burden of disease as measured in disability-adjusted life years (Gbd 2017 Risk Factor Collaborators., 2018), with psychosis and cognitive impairments representing consequences invoked as a result of high-potency cannabis use (Colizzi and Bhattacharyya, 2017; Di Forti et al., 2019). Moreover, sustained Δ9-THC exposure has been shown to drive dependence in a non-negligible proportion of users, estimated around 6–7% (Degenhardt et al., 2013), and tolerance phenomena (Colizzi and Bhattacharyya, 2018a), by inducing neurobiological alterations in brain regions relevant to addiction (Zehra et al., 2018). Finally, a fifth reason is related to the licit production of cannabis for medicinal purposes, which has increased considerably over the last 20 years, growing from 1.4 tons in 2000, mainly for purposes of scientific research, to 211.3 tons by 2016 (International Narcotics Control Board [INC], 2019). Consistent with this, several high-income countries have implemented medicinal programs with cannabis-related medicinal products for a wide range of conditions. Sativex, a cannabis plant–derived oral spray containing Δ9-THC and CBD in a 1:1 ratio, is licensed for the treatment of multiple sclerosis spasticity in Europe, Canada, Australia, Brazil, and Israel and prescribed for chronic pain. Epidiolex, a cannabis plant–derived oral CBD solution, is licensed in the United States and Europe for treatment-resistant severe forms of childhood epilepsy. Dronabinol and nabilone, synthetic compounds chemically similar to Δ9-THC, are licensed in the United States and Europe for weight loss associated with anorexia in AIDS and suboptimally controlled chemotherapy-related nausea. Estimated effectiveness of these medicinal products for the indexed indication is low (Epidiolex, dronabinol) to moderate (Sativex) (Freeman et al., 2019). Currently, clinical trials are evaluating the effectiveness of these products for different neuropsychiatric conditions, including Tourette syndrome, anxiety disorders, posttraumatic stress disorder, Alzheimer disease, Parkinson disease, psychosis, and schizophrenia (Whiting et al., 2015; National Academies of Sciences, 2017; Davies and Bhattacharyya, 2019).

Cannabis and its main ingredients have been implicated both in the development and worsening as well as in the treatment of psychosis and cognitive dysfunction. This article aims to disentangle the cannabinoid profile of different cannabis varieties with psychotogenic and intoxicating effects from that of preparations with potential therapeutic properties.

## Methods

This narrative review selectively focuses on the role of different cannabinoids in modulating psychosis and cognition. A literature search was performed using electronic databases (MEDLINE, Web of Science, and Scopus), without any time period limits, using a combination of search terms describing cannabinoids (Δ9-THC, CBD) and cognition (cognitive dysfunction/impairment/performance) or psychosis (psychotic disorder/symptoms/risk). In addition, any relevant research evidence that was not identified by this literature search has also been summarized, if considered appropriate by all authors.

### Cannabis, Psychosis, and Cognitive Dysfunction

One main issue about the association between cannabis and psychosis is its nature, namely, whether it reflects a causal relationship (Colizzi and Murray, 2018). In this perspective, longitudinal studies evaluating whether cannabis use leads to subsequent development of psychosis are of particular interest. Of 13 studies conducted so far, 10 support an increased risk of subsequently developing psychosis among cannabis users (Andreasson et al., 1987; Tien and Anthony, 1990; Arseneault et al., 2002; van Os et al., 2002; Weiser et al., 2002; Zammit et al., 2002; Fergusson et al., 2003; Ferdinand et al., 2005; Henquet et al., 2005; Manrique-Garcia et al., 2012; Rognli et al., 2015; Bechtold et al., 2016). Three more studies find a trend in the same direction failing to reach statistical significance, possibly because of short follow-up periods (Wiles et al., 2006; Gage et al., 2014) or limited sample power (Rossler et al., 2012).

Some of these studies indicate a higher likelihood of developing psychosis as a function of frequent cannabis use, a good proxy for increasing Δ-9-THC exposure (Tien and Anthony, 1990; Arseneault et al., 2002; Zammit et al., 2002; Henquet et al., 2005; Wiles et al., 2006), also confirmed by meta-analytic work (Marconi et al., 2016). Moreover, escalation of cannabis use in the immediate 5-year premorbid period increases the risk of psychosis onset (Kelley et al., 2016), with daily and high-potency cannabis use accounting, at least in part, for the higher incidence of psychosis found in some European countries (Di Forti et al., 2019). Also, while patients who stop using cannabis have the most favorable course of illness (Colizzi et al., 2016a), daily and high-potency cannabis use has been associated with higher (Schoeler et al., 2016b) and dose-dependent (Schoeler et al., 2016c) risk of psychosis relapse. Finally, magnetic resonance imaging (MRI) studies support a substantial overlap between the structural (Lorenzetti et al., 2015), functional (Blest-Hopley et al., 2018, 2019a,b), neurochemical (Sneider et al., 2013; Colizzi et al., 2016b; Blest-Hopley et al., 2019c), and structural connectivity (Rigucci et al., 2016) alterations observed upon frequent or high-potency cannabis use and those involved in the pathophysiology of schizophrenia (Howes et al., 2015).

Despite the evidence of a prospective association between cannabis and psychosis, it is important to highlight that alternative explanations for such association have been proposed, including the possibility that it might be accounted, at least partially, by the confounding effect of sociodemographic characteristics, preexisting psychiatric conditions, other substance use, self-medication, and shared genetic vulnerability (Colizzi and Bhattacharyya, 2020). Drawing conclusions as to whether the observed association represents a cause–effect relationship between exposure and disease is difficult (Castle, 2013; Ksir and Hart, 2016). According to epidemiological criteria to infer causality, cannabis use may be a component cause of psychosis (Castle, 2013; Colizzi and Bhattacharyya, 2020). In particular, the association appears to be of a modest strength, with the risk of psychosis being higher in heavy users carrying specific genetic or neurophysiological vulnerability, while most cannabis users do not develop psychosis (Castle, 2013; Ksir and Hart, 2016; Colizzi and Bhattacharyya, 2020).

The long-term effect of cannabis on cognition has been debated even more, because of inhomogeneous impairment across cognitive domains (Schoeler et al., 2016a; Lovell et al., 2019), genetically determined dose–response interindividual variability (Taurisano et al., 2016), and tolerance phenomena (Colizzi and Bhattacharyya, 2018a; Colizzi et al., 2018a, b). As for psychosis, evidence indicates a relationship between frequent (Meier et al., 2012) and high-potency (Colizzi and Bhattacharyya, 2017) cannabis use and the degree of cognitive impairments, supporting a cumulative adverse effect of Δ9-THC. This is particularly relevant to youth, because of more severe effects on a brain still in development (Meier et al., 2012; Blest-Hopley et al., 2018, 2019a; Hurd et al., 2019). However, cannabis use seems to have a modest overall impact on cognition (Scott et al., 2018), with the risk of more pronounced disrupting effects being higher in heavy users with specific biological and behavioral vulnerabilities (Jackson et al., 2016), while the effects are of limited clinical relevance for most individuals (Scott et al., 2018) and generally not enduring following abstinence (Schreiner and Dunn, 2012).

The exact mechanisms underlying the adverse effects of Δ9-THC and its interaction with other cannabinoids present in cannabis used recreationally remain unclear. In fact, the cannabis plant can produce at least 144 cannabinoids, whose effects are mostly unknown (Hanuš et al., 2016). In this regard, controlled experiments administering Δ9-THC and other cannabinoids to healthy people are particularly valuable. When implemented in an MRI design, such challenge studies may elucidate how different cannabinoids modulate human behavior by tracking the acute modulation of related neurobiological processes and their genetic, neurophysiological, and neuroreceptor determinants (Bhattacharyya et al., 2012a, b, 2014, 2017, 2018a).

### Human Laboratory Studies on Cannabinoids and Behavior

The most compelling evidence supporting a role of cannabinoids in modulating human behavior comes from experimental studies with Δ9-THC and CBD (Bhattacharyya et al., 2010). Δ-9-Tetrahydrocannabinol can induce transient (D’Souza et al., 2004; Bhattacharyya et al., 2012b, 2015a; Colizzi et al., 2019b) and less frequently persistent psychotic symptoms needing clinical attention (D’Souza et al., 2016) in otherwise healthy individuals and worsen clinical presentation in psychosis patients (D’Souza et al., 2005). Such psychosis-inducing effect is time locked to drug administration and often occurs at the same time of a transitorily impaired cognitive functioning (Curran et al., 2002; Colizzi and Bhattacharyya, 2018b), due to perturbation of underlying brain activity (Bhattacharyya et al., 2009, 2012c). Δ-9-Tetrahydrocannabinol, being a partial agonist at cannabinoid receptor 1 (CB1) (Pertwee, 2008), a potential neurobiological mechanism for its adverse behavioral effects, resides on its ability to exert a CB1-mediated facilitatory effect on striatal and prefrontal dopaminergic neurotransmission (Sami et al., 2015), possibly through a disruption of glutamate signaling (Colizzi et al., 2019a). This is in line with evidence for dopamine–glutamate aberrant interactions in psychosis and related cognitive dysfunction (Howes et al., 2015).

While Δ9-THC has shown moderate affinity for the CB1 receptor (Pertwee, 2008), synthetic cannabinoids have higher affinity, also showing full agonist action (Cohen and Weinstein, 2018). Consistent with this, risk of severe acute (Papanti et al., 2013; Castaneto et al., 2014) and long-lasting psychotic reactions for such compounds is much higher compared to Δ9-THC (Fattore, 2016; Murray et al., 2017). This is relevant, as synthetic cannabinoid recreational use has increased considerably over the last decade (Law et al., 2015).

In line with evidence that low-potency cannabis varieties with a more balanced Δ9-THC:CBD ratio are less harmful in terms of psychosis risk (Di Forti et al., 2015) and relapse (Schoeler et al., 2016b), naturalistic studies have implied less prominent acute and residual cognitive impairments in high-CBD cannabis users (Morgan et al., 2010, 2012). Also, evidence is rapidly accumulating that CBD may prevent, reverse, or attenuate the Δ9-THC–induced aberrant behavior if administered before, after, or concomitantly (Colizzi and Bhattacharyya, 2017). This seems to reflect opposite neurophysiological effects of Δ9-THC and CBD on prefrontal, striatal, and amygdalar substrates of psychiatric symptoms, such as psychosis and anxiety, as well as cognitive processes, such as verbal memory, response inhibition, fear processing, and auditory and visual stimuli processing (Bhattacharyya et al., 2010, 2012c, 2015b). The question arising is whether such opposite biobehavioral effects of Δ9-THC and CBD would reflect opposite pharmacological activities on CB receptors. However, the ability of CBD to antagonize CB receptors *in vitro* (Thomas et al., 2007) was not confirmed by subsequent evidence regarding the molecular pharmacology of CBD *in vivo* (Bih et al., 2015; McPartland et al., 2015). It may be possible that CBD affects CB receptor activity *in vivo* in an indirect manner, through other molecular targets such as the regulation of intracellular calcium levels (Bih et al., 2015).

Cannabidiol concentrations needed to offset any harmful effects of Δ9-THC in healthy individuals are still unclear (Colizzi and Bhattacharyya, 2017), and limited evidence suggests that CBD can exert different effects at different doses (Solowij et al., 2019). In particular, CBD seems to reduce the intoxicating effect of Δ9-THC when coadministered at the dose of 400 mg, a dose falling within tested therapeutic ranges (Davies and Bhattacharyya, 2019), while potentiating Δ9-THC–induced intoxication at the lower dose of 4 mg, a dose consistent with that allowed for non-medical use in some countries (Freeman et al., 2019). Further, the effects of cannabinoids other than Δ9-THC and CBD, which may be present at different concentrations in illicit cannabis products, are mostly unknown. This is also relevant, as for instance limited evidence indicates that pretreatment with Δ-9-tetrahydrocannabivarin, a CB1 receptor neutral antagonist, prevents some of the cognitive alterations observed following acute exposure to Δ9-THC, such as impairments in delayed verbal memory recall, while exacerbating others, such as memory intrusions (Englund et al., 2016).

### Understanding the Role of the Endocannabinoid System in Psychosis

Milestone discoveries in the understanding of the endocannabinoid system have been the identification of CB1 (Matsuda et al., 1990) and CB2 (Munro et al., 1993) receptors, as well as N-arachidonoyl-ethanolamine (AEA; anandamide) (Devane et al., 1992) and 2-arachidonoylglycerol (2-AG) (Mechoulam et al., 1995), endogenous ligands at CB receptors (Di Marzo and De Petrocellis, 2012). Derivatives of the arachidonic acid, AEA is a partial agonist at CB1 and CB2 receptors, whereas 2-AG is a full agonist (Di Marzo and De Petrocellis, 2012), with both showing generally lower affinity for CB receptors than Δ9-THC (McPartland et al., 2007). Both endocannabinoids are produced on demand, and their metabolites, obtained through enzymatic hydrolysis, show biological properties (Kano et al., 2009).

Cannabinoid receptor 1 receptor signaling in the brain is essential to modulate neurotransmitter release (Colizzi et al., 2016b) and maintain neuronal activity in a balanced regimen (Zou and Kumar, 2018). The evidence that Δ9-THC, CBD, and potentially other cannabis plant–derived cannabinoids may modulate CB1 receptor in the brain makes them competitors of the endocannabinoids at the same receptor, with important implications for the homeostasis of the endocannabinoid system (Pertwee, 2008). An altered endocannabinoid signaling has been independently implied in psychosis (Lu and Mackie, 2016) from investigations of central nervous system biomarkers, suggesting ubiquitously higher CB1 receptor binding in the brain, lower levels of CB1 messenger RNA and protein in the prefrontal cortex, higher prefrontal metabolism of 2-AG, and elevated AEA levels in the cerebrospinal fluid (Volk and Lewis, 2016; Minichino et al., 2019), as well as evidence for higher AEA peripheral blood concentrations and higher CB1 receptor expression on peripheral immune cells (Minichino et al., 2019). Very recent evidence indicates elevated endocannabinoid levels even in the peripheral blood of people at clinical high risk (CHR) of psychosis (Appiah-Kusi et al., 2019), that is, people presenting with prodromal or subsyndromal psychotic symptoms suggestive of a prepsychotic phase or attenuated psychosis syndrome (Fusar-Poli et al., 2013), thus suggesting a perturbation of the endocannabinoid system in the early phases of the disorder. It is worth mentioning that studies measuring endocannabinoid levels in both the brain parenchyma and peripheral blood did not find any correlation between the alterations observed in the two body compartments (Minichino et al., 2019). Further studies are needed to investigate whether they are independently associated with psychosis.

### Cannabis-Based Potential Treatments for Psychosis and Cognitive Dysfunction

Evidence that Δ9-THC and other direct-acting cannabinoid agonists can induce psychotic symptoms in both healthy individuals (Colizzi et al., 2019b) and psychosis patients (Henquet et al., 2010), and hyperactivity of the endocannabinoid system may independently promote the developmental cascade toward psychosis (Lu and Mackie, 2016), fueled the study of CB1 receptor antagonist potential in schizophrenia. Unfortunately, evidence on the efficacy of such novel compounds was disappointing (Meltzer et al., 2004). Studies also revealed important side effects of CB1 receptor antagonists/inverse agonists, including provoking mood alterations and suicidal ideation (Janero and Makriyannis, 2009). A promising strategy to improve the pharmacological and safety profile of CB receptor blockers is to shift from orthosteric to allosteric ligands. While orthosteric therapeutic compounds would compete with endocannabinoids at the CB1 receptor until metabolized, allosteric compounds would selectively target distinct CB1 receptor allosteric binding site(s), modulating the effect of endocannabinoids or other orthosteric ligands, such as Δ9-THC, only when and where active (Dopart et al., 2018).

In this respect, CBD has also been suggested to be a non-competitive CB1 receptor antagonist, with low affinity for its primary ligand site but negative allosteric modulation properties allowing it to alter the potency of other primary ligands such as endocannabinoids and Δ9-THC in a dose-dependent manner (Laprairie et al., 2015). Despite lacking intrinsic efficacy, CBD would modulate the endocannabinoid tone, reducing CB1 receptor activity in the absence of the side effects previously found in CB1 inverse agonist trials (Chye et al., 2019), thus representing a promising CB receptor blocker. However, this potential mechanism of action does not exclude the possibility of an indirect modulation of the endocannabinoid system mediated by other molecular targets that, in conjunction with the allosteric binding, would contribute to the overall effects observed *in vivo*.

Not surprisingly, the antipsychotic potential of CBD has been the subject of evaluation since the 1990s, with 13 studies conducted so far (Davies and Bhattacharyya, 2019). Such studies widely differ in terms of study design (open-label, placebo-controlled, comparative treatment, add-on treatment), sample size (*N* = 1–88), CBD dosage (300–1,500 mg/d), length of treatment (single dose, 6 months), psychiatric condition (schizophrenia, psychosis in Parkinson disease, CHR), and outcome measure (psychotic symptoms, psychosocial functioning, stress response, functional MRI, and cognitive processing). Early open-label case report, case series, and pilot studies indicate that a 4-week treatment with CBD reduces psychotic symptoms in schizophrenia (Zuardi et al., 1995) and Parkinson disease (Zuardi et al., 2009), but not in bipolar disorder (Zuardi et al., 2010), also reducing symptom severity in 1 of 3 patients with treatment-resistant schizophrenia (Zuardi et al., 2006). The first clinical trial providing solid evidence for CBD antipsychotic properties as monotherapy allocated schizophrenia patients to either CBD or the antipsychotic amisulpride up to 800 mg/d for 4 weeks, proving non-inferiority of CBD in reducing psychotic symptoms, with the advantage of a better tolerability profile (Leweke et al., 2012). Such effect of CBD in reducing psychotic symptoms was not confirmed in a subsequent study where CBD was administered at the lower dose of 600 mg/d for 2 weeks only (Leweke et al., 2014). More recently, a placebo-controlled study supported the efficacy of 1,000 mg/d CBD as add-on treatment in producing additional positive psychotic symptom reduction and overall clinical improvement in schizophrenia patients on an antipsychotic regimen for 6 weeks, with adverse events similar to placebo (McGuire et al., 2018). Another study implementing the same methodology did not replicate such add-on effect of CBD at the lower dose of 600 mg/d in an older population of schizophrenia patients receiving long-term polypharmacy (Boggs et al., 2018). Besides confirming a potential threshold dose–response curve, where higher CBD doses would be needed to reach antipsychotic effect (Crippa et al., 2018), it also raises the questions whether CBD may be involved in drug-to-drug interactions whose effects are unclear and whether younger patients may benefit more from CBD treatment, because of earlier intervention in the pathophysiology of psychosis. Consistent with this, recent evidence supports the efficacy of a single dose of 600 mg CBD in normalizing aberrant brain function underlying psychotic symptoms in antipsychotic medication-naive CHR individuals (Bhattacharyya et al., 2018; Wilson et al., 2019) and early psychosis patients (O’Neill et al., 2020). Also, additional evidence indicates that a 7-day treatment with 600 mg/d CBD may partially attenuate the altered responses to stress observed in CHR individuals (Appiah-Kusi et al., 2020). Altogether, these findings nourish hope that CBD may act as a disease-modifying drug.

Evidence for improving effects of CBD on cognition reveals a less linear dose–response effect (Davies and Bhattacharyya, 2019). In a study of cognition in schizophrenia, 1-month treatment with CBD improved selective attention at the dose of 300 mg/d, while being less effective at the higher dose of 600 mg/d, possibly because of sedative effects in the higher-dose group (Hallak et al., 2010). In another study, the same 600 mg/d regimen did not improve cognition among schizophrenia patients after 6 weeks of add-on treatment (Boggs et al., 2018). Also, an add-on dose of 1,000 mg/d, the highest ever tested for cognitive effects in psychosis, failed to improve cognition significantly in a 6-week schizophrenia trial (McGuire et al., 2018). Interestingly, a recent study indicates that a 400 mg CBD dose, while protecting against the intoxicating effects of Δ9-THC, exhibits intoxicating potential on its own in healthy individuals (Solowij et al., 2019). Limited evidence also supports the ability of low CBD doses as 16 mg to improve emotional recognition acutely when administered to cannabis users (Hindocha et al., 2015). Altogether, compared to the effects of CBD on psychosis, evidence points in the direction of a narrower and potentially bell-shaped dose–response for the effects of CBD on cognition, with enhancing effects at low doses, which diminish to the extent of inducing intoxication/impairments at higher doses (Linares et al., 2019).

## Discussion

**FIGURE 1:**
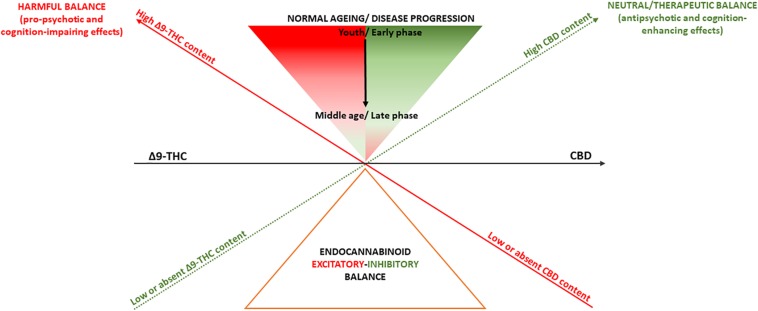
Summary of the effects of Δ9-THC and CBD on psychosis and cognition. A deliberately simplistic interpretation of findings presented here is that cannabis-derived cannabinoids Δ9-THC and CBD, through their almost opposite partial agonist/agonist and antagonist/inverse agonist activity, might respectively, induce or blunt endocannabinoid system hyperactivity, resulting in pro-psychotic or antipsychotic effects as well as cognition-impairing or cognition-enhancing effects. However, despite producing opposite actions on the endocannabinoid system, Δ9-THC and CBD may exhibit even similar effects as activating or inhibiting the endocannabinoid system may produce both symptom amelioration and worsening depending on aging as well as different phases of psychosis or cognitive deterioration.

The endocannabinoid system modulates a wide range of biological processes through life, ranging from neurodevelopment to neurodegeneration (Di Marzo et al., 2015). It is thus plausible that pharmacological manipulation of the endocannabinoid signaling, depending on the direction of its effects (Pertwee, 2008), may have either deleterious consequences or therapeutic advantages. Consistent with this, depending on their Δ9-THC:CBD ratio, cannabis-derived drugs may have both pro-psychotic and antipsychotic as well as cognition-impairing and cognition-enhancing effects (Lu and Mackie, 2016; Figure 1). However, it is important to note that such model to explain the effects of cannabis on psychosis and cognition does not necessarily apply to other medical conditions. For instance, evidence points in the direction of a potential therapeutic role of Δ9-THC in multiple sclerosis spasticity, chronic pain management, weight loss associated with anorexia in AIDS, and chemotherapy-related nausea (Freeman et al., 2019).

Also, the endocannabinoid system function may change physiologically because of normal aging or be affected earlier in life in response to a neuropsychiatric condition and differently depending on its phases (Di Marzo et al., 2015). This has implications for the homeostasis of other neurotransmitter systems, such as glutamate and dopamine, which also go through dynamic changes in health (Kaiser et al., 2005; Rothmond et al., 2012) and disease (Howes et al., 2015). It is therefore not unreasonable to speculate that Δ9-THC and CBD effects may vary depending on patients’ aging and disease progression (Di Marzo et al., 2015). Limited preclinical evidence suggests that low Δ9-THC doses may reverse the age-related decline in cognitive performance, while still impairing performance in youth (Bilkei-Gorzo et al., 2017). On the other hand, CBD does not seem to produce additional benefit as add-on treatment for psychosis patients in their middle age (≥45 years) (Boggs et al., 2018), while ameliorating psychosis and tending to improve cognition (McGuire et al., 2018), as well as normalizing underlying neurophysiological processes (Bhattacharyya et al., 2018b) in earlier phases of the disorder (Figure 1).

Overall, evidence discussed here provides clarification for the multifaceted effects of cannabis on psychosis and cognition, by also navigating the complex role of the endocannabinoid system in both the harmful and therapeutic effects of cannabis-related products. These considerations provide a stepping-stone to the development of cannabinoid treatments for symptom amelioration and disease modification in psychosis. However, despite being promising, research in this field is still in its infancy, and we are far from clear-cut evidence that cannabinoids have a therapeutic role in psychosis or any other mental disorder (Black et al., 2019). Future research will need to optimize the pharmacological manipulation of the endocannabinoid signaling, before any cannabis-related medical product for the treatment of psychosis and cognitive impairment might actually make it to the market.

## Author Contributions

All authors co-wrote and edited the manuscript. MC provided leadership for decisions of content, framing, and style and led the creation of the Figure.

## Conflict of Interest

The authors declare that the research was conducted in the absence of any commercial or financial relationships that could be construed as a potential conflict of interest.
